# A Comparative Study of Particle Size Distribution of Graphene Nanosheets Synthesized by an Ultrasound-Assisted Method

**DOI:** 10.3390/nano9020152

**Published:** 2019-01-26

**Authors:** Juan Amaro-Gahete, Almudena Benítez, Rocío Otero, Dolores Esquivel, César Jiménez-Sanchidrián, Julián Morales, Álvaro Caballero, Francisco J. Romero-Salguero

**Affiliations:** 1 Departamento de Química Orgánica, Instituto Universitario de Investigación en Química Fina y Nanoquímica, Facultad de Ciencias, Universidad de Córdoba, 14071 Córdoba, Spain; q22amgaj@uco.es (J.A.-G.); q12esmem@uco.es (D.E.); qo1jisac@uco.es (C.J.-S.); 2 Departamento de Química Inorgánica e Ingeniería Química, Instituto Universitario de Investigación en Química Fina y Nanoquímica, Facultad de Ciencias, Universidad de Córdoba, 14071 Córdoba, Spain; q62betoa@uco.es (A.B.); b42otizr@uco.es (R.O.); iq1mopaj@uco.es (J.M.)

**Keywords:** graphene nanosheets, exfoliation, particle size distribution, nanoparticle tracking analysis, asymmetric flow field flow fractionation

## Abstract

Graphene-based materials are highly interesting in virtue of their excellent chemical, physical and mechanical properties that make them extremely useful as privileged materials in different industrial applications. Sonochemical methods allow the production of low-defect graphene materials, which are preferred for certain uses. Graphene nanosheets (GNS) have been prepared by exfoliation of a commercial micrographite (MG) using an ultrasound probe. Both materials were characterized by common techniques such as X-ray diffraction (XRD), Transmission Electronic Microscopy (TEM), Raman spectroscopy and X-ray photoelectron spectroscopy (XPS). All of them revealed the formation of exfoliated graphene nanosheets with similar surface characteristics to the pristine graphite but with a decreased crystallite size and number of layers. An exhaustive study of the particle size distribution was carried out by different analytical techniques such as dynamic light scattering (DLS), nanoparticle tracking analysis (NTA) and asymmetric flow field flow fractionation (AF4). The results provided by these techniques have been compared. NTA and AF4 gave higher resolution than DLS. AF4 has shown to be a precise analytical technique for the separation of GNS of different sizes.

## 1. Introduction

Particle size analysis is a key element because many properties of nanomaterials are size dependent [1]. This parameter is essential since the synthesis at a small scale must be monitored for subsequent bulk production and for the control of nanotechnological products in the market. The control of size during the synthesis of nanomaterials is decisive in various industrial sectors such as nanomedicine, nanofood, nanoenergy or nanocosmetics [2,3,4,5,6].

It is well known that graphene has excellent properties that provide a multitude of technological applications in different fields. It is formed by a pattern of hexagonal rings of carbon atoms constituting a huge flat molecule [7]. Some of its most important properties are its high thermal and electrical conductivity, its high elasticity, its hardness and strength and its flexibility, in that it is more flexible than carbon fibre and equally lightweight [8]. 

Many methods for obtaining graphene have been reported. Graphene layers can be obtained by liquid-phase exfoliation of commercial graphite in different organic solvents using the ultrasonic technique. This method requires special experimental conditions at which very high pressures and temperatures are reached in very short periods of time [9]. The colloidal graphene generated has the same surface energy as the solvent used. In this way, the free energy is negative, and the structure is broken, giving rise to fragments of graphite interspersed with solvent molecules [10]. For the successful exfoliation of graphite, different organic solvents have been used, such as N-methyl pyrrolidone (NMP), dimethylformamide (DMF), pyridine and *o*-dichlorobenzene (ODBC) [11,12]. By means of the ultrasound method, a maximum of 15% of individual graphene sheets is obtained. The rest is a mixture of multilayer graphene or few-layer graphene. A very important parameter for obtaining few-layer graphene is to carry out a selective centrifugation [13]. By carrying out this operation it is possible to separate the non-exfoliated graphitic particles from the exfoliated graphene sheets. This method of synthesis has different advantages with respect to conventional graphene production methods: strong oxidizing agents are not required, the synthesis time is reduced and unfunctionalized and non-oxidized graphenes are obtained in one step [14]. On the contrary, this process can have negative effects, since prolonged sonication times can facilitate the existence of defects in the surface of the graphene sheets and the reduction of the layer size [15]. For instance, these problems have been observed when DMF is used as an exfoliating liquid phase [16]. For this reason, it is very important to control the sonication times during the exfoliation process of the graphite and, above all, to choose the solvent that generates the least problems [17].

*o*-Dichlorobenzene is an organic solvent that has excellent properties to be used as liquid phase for the process of graphite exfoliation. It is commonly applied as a reaction medium in fullerene chemistry and it is well known that it forms very stable dispersions due to its very efficient interactions with graphene via π-π stacking [18]. On the other hand, it has a high boiling point and a surface tension suitable for the correct exfoliation [12]. 

Graphene-based materials have been characterized by a variety of different techniques, such as X-ray diffraction, transmission electron microscopy and Raman spectroscopy, among others [19,20]. Although these techniques can provide some information about the size of these materials, dynamic light scattering (DLS) is particularly useful to determine particle size. Thus, the use of DLS to measure the size of graphene nanosheets and exfoliated graphene oxide has been reported as a simple and fast method of characterization [21]. This technique is based on the Brownian motion of the particles in suspension causing the scattering of light at different time-dependent fluctuations intensities. These fluctuations are directly related to the diffusion coefficient of the particles in the solvent, which provides in turn the particle size using the Stokes-Einstein relationship. However, it is well known that this technique is more reliable for spherical particles than for non-spherical particles [22]. Moreover, if a sample is polydisperse, as usually happens for graphene nanomaterials prepared by most methods, DLS will exhibit some limitations to measure particle size (vide infra) [23]. Furthermore, two techniques have emerged in the last years for the characterization of nanoparticles, i.e., nanoparticle tracking analysis (NTA) and asymmetric flow field-flow fractionation (AF4). To the best of our knowledge, none of them has been used for the characterization of graphene-based materials.

NTA is a relatively novel technique for the study of particle size distribution. Like DLS, NTA uses light scattering and the Brownian motion of liquid suspensions of particles to obtain the size distribution in a sample. This technique allows real-time visualization and recording of nanoparticles during the measurement by combining a charge-coupled device with laser light scattering microscopy [24]. In this way, the NTA software tracks individual particles and, using a formula derived from the Stokes–Einstein equation, provides particle size values by calculating the particle hydrodynamic diameter. In addition, it gives other parameters such as particle concentration, the range of which must be 10^6^ to 10^9^ particles/mL, aggregation and fluorescence [25]. The concentration of particles present in the sample does not depend on the scattered light, so this technique allows an accurate and reproducible determination of the concentration of non-spherical particles as in the case of carbon nanotubes or colloids [26]. Based on these characteristics, NTA is frequently used to accurately obtain the size of protein aggregates and drug delivery nanoparticles [27].

AF4 is one of the most promising techniques for the characterization of nanoparticles due to the high precision and wide separation range it provides, as well as the great variety of nanomaterials that can be analysed. Currently, this technique is in continuous progress and shows high versatility, because it has been used to characterize many types of nanomaterials such as nanodrugs, silica nanoparticles, nanocellulose and nanoplastics, among others [28,29,30,31]. AF4 is a chromatographic technique consisting of a laminar flow of a carrier liquid combined with a transverse flow. The induced cross-flow field interacts with any molecule or particle in the channel and, in this way, size separation occurs [32]. This phenomenon gives rise to different elution times, which are inversely proportional to the diffusion coefficients of the particles in the sample, that is, to the radius of gyration. This technique is able to separate particles with sizes ranging from 2 nm to 1 µm [33]. Furthermore, it can be coupled with different types of detectors such as UV-Vis, multiangle light scattering (MALS) and/or dynamic light scattering (DLS) that would allow the complete characterization of different nanomaterials [34,35], and the determination of the molar mass distribution of macromolecules [36]. In addition, AF4 has been coupled online with ICP-MS for the selective detection of TiO_2_ nanoparticles [37] and in combination with UV-Vis spectroscopy and off-line HR-ICP-MS for the determination of Ag nanoparticles [38], rendering this technique very useful and versatile for analysing different types of materials.

Herein, we report the synthesis of unfunctionalized, non-oxidized and isolated graphene nanosheets (GNS) applying the ultrasound-assisted method by liquid-phase exfoliation of a micrographite. The nanosheets obtained have been characterized using different techniques, particularly related to the study of particle size. Thus, besides XRD, TEM and Raman spectroscopy, three techniques, specifically oriented to the determination of particle size, i.e., DLS, NTA and AF4, were investigated. The proper choice of the parent micrographite has been essential because it has provided GNS particles measurable by all these techniques. A comparative study of the results obtained with the different characterization techniques is reported. 

## 2. Materials and Methods 

### 2.1. Materials 

Microcrystalline graphite powder (98%) was purchased from Nanostructured & Amorphous Materials Inc. (Katy, TX, USA). 1,2-dichlorobenzene (ODCB, anhydrous, 99%), dichloromethane (anhydrous, ≥99.8%, contains 40–150 ppm amylene as stabilizer) and lanthanum hexaboride (powder, 10 μm, 99%) were obtained from Sigma Aldrich (San Luis, MO, USA).

### 2.2. Methods

#### 2.2.1. Synthesis of Graphene Nanosheets (GNS)

250 mg of microcrystalline graphite powder (MG) were stirred in 50 mL of *o*-dichlorobenzene (ODCB) for 30 min. Subsequently, the resulting dispersion was subjected to treatment with a pulsed ultrasound probe for 2 h using an Ultrasonic Homogenizer 4710 Series from Cole Parmer Instrument Co. (Vernon Hills, IL, USA) (45% amplitude, 60% duty cycle). The power supplied by the equipment was 9.96 W, according to a calorimetric calibration method [39] (see SI and Figure S1). The suspension obtained was centrifuged at 1935× *g* (4000 rpm) for 30 min to remove the non-exfoliated micrographite. The remaining suspension was again centrifuged at 12,096× *g* (10,000 rpm) to isolate the graphene nanosheets. They were washed three times with dichloromethane by successive redispersions and centrifugations to remove the ODCB. A Sorvall Super T-21 Tabletop Superspeed Centrifuge (Du Pont, Delaware, NJ, USA) was used to carry out all centrifugation processes. Dichloromethane was evaporated from the resulting graphene nanosheets dispersion at room temperature for 12 h and, finally, the powdered exfoliated GNS were dried under vacuum at 80 °C overnight.

#### 2.2.2. X-ray Diffraction Analysis (XRD)

X-ray diffraction patterns of the micrographite and graphene nanosheets were performed using a Bruker D8 Discover (Billerica, MA, USA) with a monochromatic CuKα radiation source. The scanning conditions for structural analysis were an angular range of 15°–80° (2θ), a 0.040° step size and 1.05 s per step. Additional measurements of X-ray diffraction were carried out using a Bruker D8 Advance. The scanning condition for this analysis were an angular range of 5°–40° (2θ), a 0.002° step size and 9 s per step.

#### 2.2.3. Transmission Electron Microscopy 

Transmission electron microscopy (TEM) images were recorded on a Jeol JEM-1400 transmission electron microscope (Akishima, Tokyo, Japan) operated at an accelerating voltage of 120 kV. The instrument has a high-contrast objective-lens polepiece, enabling high-contrast TEM image observation at all magnifications and good image quality, as well as easy handling. In addition, it has an advanced vacuum system. The digital-camera configuration includes a CCD camera that enables focusing and image verification on the operation screen. The measurement CCD camera (MC), orthogonal to the alignment camera, has a resolution of 1344 × 1024 pixel (12 bits/pixel). The measurements have been made in a magnification range between 8000× and 150,000× with an instrument resolution of 0.38 nm between points.

#### 2.2.4. Raman Spectroscopy

Raman spectra were acquired with a Renishaw Raman instrument (InVia Raman Microscope, Wotton-under-Edge, UK) equipped with a Leica microscope furnished with various lenses, monochromators and filters in addition to a CCD camera. A silicon standard sample was used as reference for calibration (520 cm^−1^). Spectra were obtained by excitation with green laser light (532 nm) from 150 to 3500 cm^−1^. A total of 32 scans per spectrum were acquired to improve the signal-to-noise ratio.

#### 2.2.5. X-ray Photoelectron Spectroscopy (XPS)

X-ray photoelectron spectroscopy (XPS) was performed through a SPECS mod. PHOIBOS 150 MCD spectrometer (Berlin, Germany) using non-monochromatic Mg Kα radiation and a multichannel detector. All spectra were fitted to Gauss–Lorentz curves to better identify the different chemical environment of the elements in each material. The binding energy values were calibrated with the adventitious carbon C 1s signal (284.8 eV).

#### 2.2.6. Dynamic Light Scattering (DLS)

Particle sizes were measured in a Zetasizer ZSP (Malvern Instrument Ltd., Worcestershire, UK) at 25 °C based on laser Doppler velocimetry and dynamic light scattering (DLS) techniques. Previously, the suspension was homogenized using an ultrasonication probe for a period of 5 min. 

The refraction index values were set at 1.33 and 2.38 for the dispersant (deionized water) and the material (carbon), respectively. The analysis was carried out by triplicate and medium and standard deviation were calculated. To obtain the hydrodynamic radius (R_h_) of the GNS particles, the hydrodynamic diameter (D_h_) was calculated by using the Stokes–Einstein Equation (1):

(1) $$D_{h}=\frac{K_{B\;}T}{{3\pi \eta }_{0}D_{t}}$$

where K_B_ is the Boltzmann constant, T the temperature in K degrees, $\eta_{0}$ the solvent viscosity, and D_t_ the translational diffusion coefficient.

The intensity size distribution or the Z-average diameter was obtained from the autocorrelation function using the “general purpose mode” for the materials.

#### 2.2.7. Nanoparticle Tracking Analysis (NTA)

NTA measurements were performed in a NanoSight NS300 (Malvern Panalytical Ltd., Malvern, UK), equipped with a sample chamber and a 488 nm laser.

For the NTA measurements, 1 mg of GNS was suspended in 1 mL of water. Before the analysis, the solution was homogenized for 3 min in an ultrasonic bath. The samples were injected into the chamber with sterile syringes. Many individual particles are tracked during their Brownian motion by the software, which uses the Stokes–Einstein Equation (2) to provide sample information:

(2) $${\bar{\left(x,y\right)}}^{2}=\frac{{2K}_{B}T}{{3R}_{h}\pi \eta }$$

where K_B_ is the Boltzmann constant and ${\bar{\left(x,y\right)}}^{2}$ is the mean-squared speed of a particle with a hydrodynamic radius of R_h_ in a medium of defined viscosity and temperature. All measurements were performed at room temperature.

#### 2.2.8. Asymmetric Flow Field Flow Fractionation (AF4)

An AF4 system (AF2000, Postnova Analytics, Landsberg am Lech, Germany) coupled online to a 21-angle MALS detector (PN3621, Postnova Analytics, Landsberg am Lech, Germany) and UV-Vis diode array detector (PN3241 Postnova Analytics) was used. The MALS detectors were calibrated using bovine serum albumin monomer and detectors at different angles were normalized with respect to a 90° detector measuring a sodium poly(styrenesulfonate) (PSS) standard. The AF4 channel was trapezoidal-shaped, 350 μm thick (defined by a spacer), 29.8 cm long (inlet to outlet) and had a maximum width of 2 cm. Particle recoveries over AF4 were tested with several commonly used membranes. The analytical parameters and characteristics of our AF4 system are presented in Table 1. All injections were carried out with an autosampler in triplicate (PN5300, Postnova Analytics).

## 3. Results

### 3.1. X-ray Diffraction (XRD)

**Figure 1:**
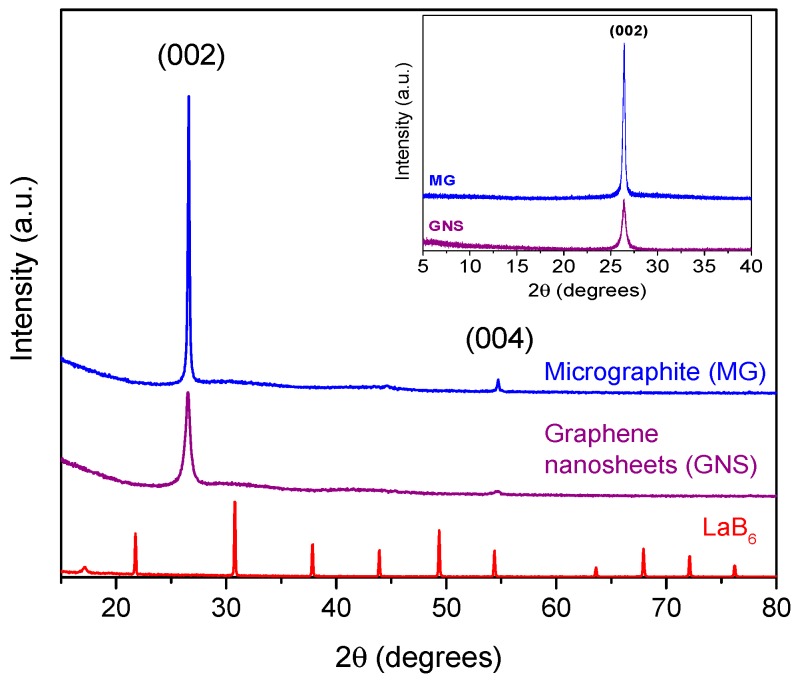
XRD patterns of micrographite (MG), graphene nanosheets (GNS) and LaB6 samples. Inset: XRD patterns of MG and GNS recorded from 5° to 40° (2θ).

The structural properties of micrographite (MG) and graphene nanosheets (GNS) samples were examined by XRD. Figure 1 shows the XRD patterns, which revealed the characteristic peaks for these materials. Both samples exhibited a strong peak at ca. 26.5° (2θ), which corresponded to the (002) graphite reflection [40]. Also, a weak peak at 54.5° (2θ), attributed to the (004) reflection, was present in the samples, especially in the micrographite. The absence of the graphite peak for graphene oxide (GO) at 11° (2θ) [41] confirmed that the graphene nanosheets preparation method did not cause partial oxidation of the starting graphite, as shown in the inserted image in Figure 1. The higher relative intensity of the graphite signals indicated a higher crystallinity compared to the GNS. The full widths at half-maximum of the (002) peak (FWHM) were studied to determine the crystallite size (LaB_6_ was used as a pattern to determine the instrumental broadening). FWHM for GNS was higher than for MG (see Table 2). The crystallite size, L, was calculated through the Scherrer’s Equation (3):

(3) $$L=\frac{k\;\lambda }{\beta \cos\theta }$$

where λ is the X-ray wavelength in nanometer (nm), β is the peak width of the diffraction peak profile at half maximum height in radians, θ is the scattering angle in radians and k is a constant related to crystallite shape, taken as 0.9 for the (002) Bragg reflection.

The instrumental broadening effect on FWHM was subtracted out using Warren’s method on the assumption of a Gaussian peak [42]:

β^2^ = β^2^_sample_ − β^2^_instrumental_ (4)

where β_instrumental_ is referred to LaB_6_ and equal to 0.130.

Liquid-phase exfoliation by ultrasound-assisted synthesis for obtaining graphene nanosheets (GNS) produced smaller crystallite sizes. Moreover, the thickness of the crystallites along c axis decreased because the full width at half-maximum (FWHM) of the peak (002) was higher than that of the starting micrographite (MG) [42,43]. Thus, the action of ultrasonic waves generates a cavitation energy that caused an increase in the interlayer spacing (d_002_) due to the exfoliation of the MG [44]. In this way, the GNS sample had a smaller crystallite size and a lower average stacking height of the layered structure, confirming an effective exfoliation of the graphite by the ultrasound probe treatment.

### 3.2. Transmission Electronic Microscopy (TEM)

**Figure 2:**
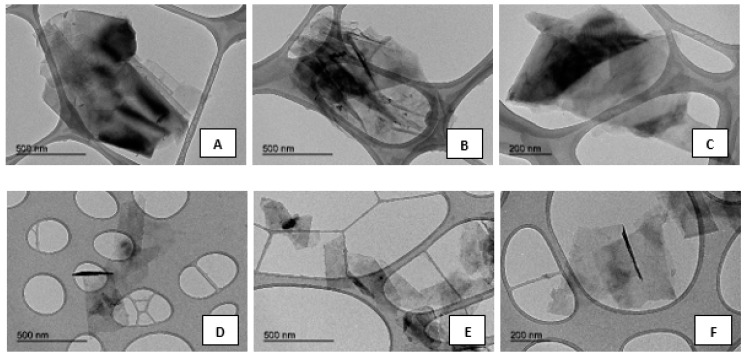
TEM images with different magnification for (**A**–**C**) micrographite (MG) and (**D**–**F**) graphene nanosheets (GNS) samples.

The surface morphology of the original graphite and the synthesized graphene was examined by TEM images. Nanometric particles of graphite flakes are shown in Figure 2A–C. Graphene nanosheets (GNS) were observed as transparent layers (see Figure 2D–F). When graphene sheets folded over themselves, cross-sectional and a few layers with nanometric size 200–500 nm could be viewed. The folded graphene sheets that were placed parallel to the electron beam were observed as several dark lines. However, TEM images for graphite showed a dark spot due to the ordered stacking of a large number of layers. Also, it was verified that the size of the graphite particles were several times larger than GNS particles [40]. 

### 3.3. Raman Spectroscopy

**Figure 3:**
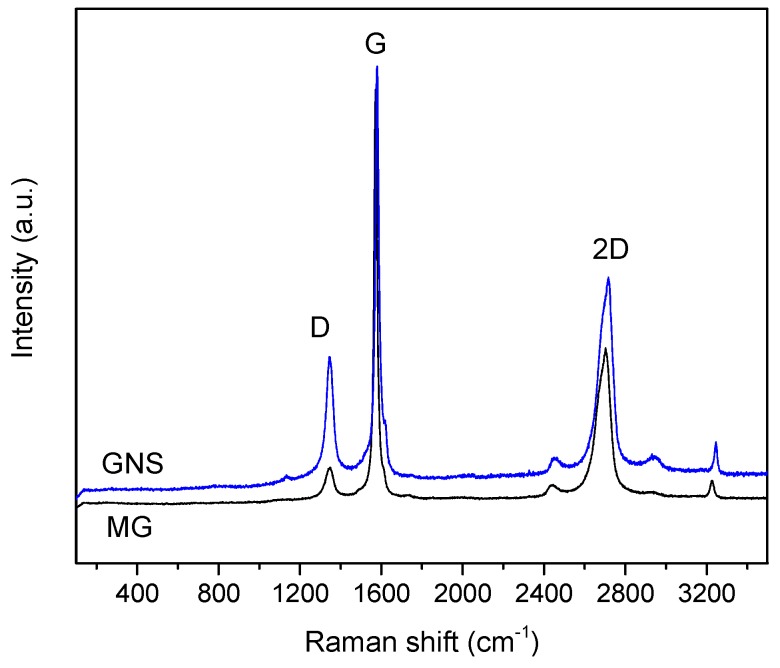
Raman spectra of MG and GNS samples.

Raman spectroscopy was performed to confirm the exfoliation of the micrographite by ultrasound irradiation. The typical bands of graphene materials were observed in the spectra (Figure 3). The G peak corresponds to the phonons of the energy level E_2g_ in the Brillouin zone. The intensity of the D band indicates the degree of disorder that the material possesses, which is related to several factors such as functionalization in the graphene macromolecular sp^2^ ring or, in our case, the ultrasonic exfoliation in ODCB. In many studies, the ratio of I_D_/I_G_ intensities is used to verify that the material has undergone a structural change during the synthesis process [45]. In addition, it has already been reported that this ratio is related to the flake size. Thus, an increase in the I_D_/I_G_ ratio indicates a smaller sheet size because the ratio of edge defects is greater for smaller layers [46]. The reference MG had I_D_/I_G_ = 0.10 while this ratio increased to 0.38 for the exfoliated GNS. These values confirmed the exfoliation of the graphite in ODCB using the ultrasound probe, which caused a certain disorder in the material. 

The calculation of the number of graphene layers is very relevant in this type of study. Many investigations relate the position of the G peak with the number of graphene layers. The G peak of the GNS was at a Raman shift of 1582 cm^−1^ and so, applying the equation proposed by Wang et al. [47], it can be deduced that this material consisted of approximately seven layers of graphene nanosheets. The 2D peak was very remarkable, which is the overtone of the peak D. The position, the shape of the line and the intensity of the peak are related to the number of layers [48]. Normally, a single layer of graphene shows a band at 2679 cm^−1^. It has been shown that in the case of multilayers graphene, the 2D peak shifts toward higher Raman shift and becomes broader [49]. In the case of GNS, the 2D band was centred at 2716 cm^−1^, similar to MG, although there was a clear difference in peak intensity between them. It is common to calculate the ratio of peak intensity I_2D_/I_G_. An approximate value of 2 identifies a single layer of graphene [50]. The original micrographite showed an intensity ratio of 0.38 while the graphene nanosheets obtained after the ultrasonication process had a higher ratio of 0.55, thus confirming the exfoliation of graphite again.

### 3.4. X-ray Photoelectron Spectroscopy (XPS)

**Figure 4:**
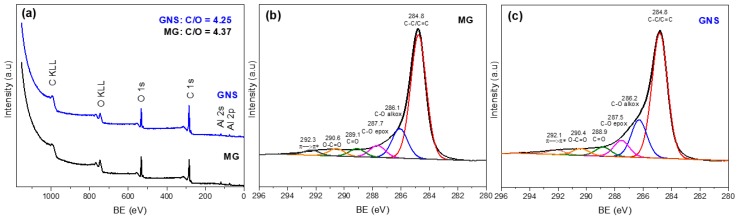
(**a**) XPS survey for MG and GNS and XPS spectra for the C 1s photoemission peak of (**b**) MG and (**c**) GNS.

In order to study the composition of MG and GNS, XPS spectra of these samples were measured. Figure 4a shows the XPS survey for the MG and GNS samples. Both materials were composed of C and O. The presence of Al is due to the holder where samples were placed. The oxygen content in the original graphite is in accordance with the purity of the sample and the presence of noncovalently bonded adsorbed oxygen [51]. The C/O ratio for GNS and MG samples, which is inserted in this figure, confirmed that the GNS material underwent a very slight oxidation upon exfoliation even though the difference between the MG and GNS ratios was almost negligible and insignificant compared to a common graphene oxide (GO) [52]. The X-ray photoelectron spectra in the C 1s region of MG (Figure 4b) and GNS (Figure 4c) were fitted by overlapped peaks attributed to C–C bonds (284.8 eV), C–O bonds in hydroxy (286.1 eV), epoxy (287.7 eV), carbonyl (289.1 eV) and carboxyl (290.6 eV) groups, and the π→π* transition (shake-up satellite peak at 292.3 eV) [53]. Therefore, no obvious changes were observed on the chemical structure of pristine MG and GNS obtained by the ultrasound-assisted method according to the C 1s spectra. Therefore, it is confirmed that this method of synthesis allows obtaining GNS directly from MG, thus avoiding the synthesis of graphitic oxide (GO). The contribution of each component is summarized in Table 3. The values obtained for both samples are very similar, concluding that this method of exfoliation of the micrographite by the ultrasound treatment causes a delamination of the pristine graphite avoiding oxidation processes. These results are in agreement with those reported for graphene nanosheets prepared via ultrasonic exfoliation of graphite powder in *N*-methyl-2-pyrrolidone [54].

### 3.5. Particle Size Distribution by DLS 

**Figure 5:**
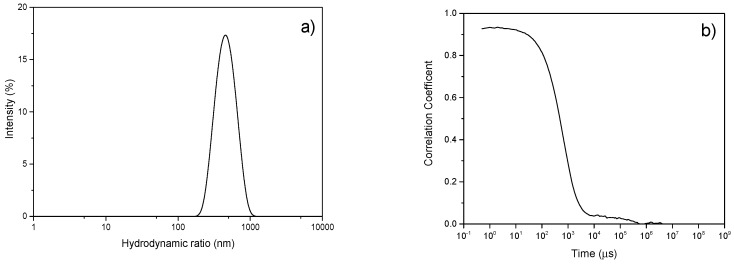
(**a**) Dynamic light scattering (DLS) size distribution curve of GNS and (**b**) normalized intensity correlation function of the scattered light intensity of GNS.

The size distribution and intensity correlation function normalized between one and zero of the GNS using dynamic light scattering (DLS) are shown in Figure 5. The advantage of applying this technique is its versatility in terms of fast, easy, reproducible and non-destructive results. DLS provides the hydrodynamic radius (R_h_), which is defined as the radius of an equivalent hard sphere diffusing at the same rate as the particle under observation, and so it is indicative of the apparent size adopted by the GNS in the aqueous dispersion. GNS exhibited a Gaussian peak located at 450 ± 14 nm. The measurement carried out for the MG material revealed a particle size outside the quantification limits of this technique (>1 μm).

DLS also provides the polydispersity index (PDI), which indicates the width of the particle size distribution, being calculated as (peak width/peak height)^2^. A value of PDI < 0.1 indicates that the sample is monodisperse. Instead, if PDI is between 0.1 < PDI < 0.2, the sample would have a narrow particle size distribution. In the case of GNS, PDI was 0.36, value between 0.2 < PDI <0.5, which indicates that the sample has a wide particle size distribution according to the results reported by J. Lohrke et al. [55].

### 3.6. Particle Size Distribution by NTA

**Figure 6:**
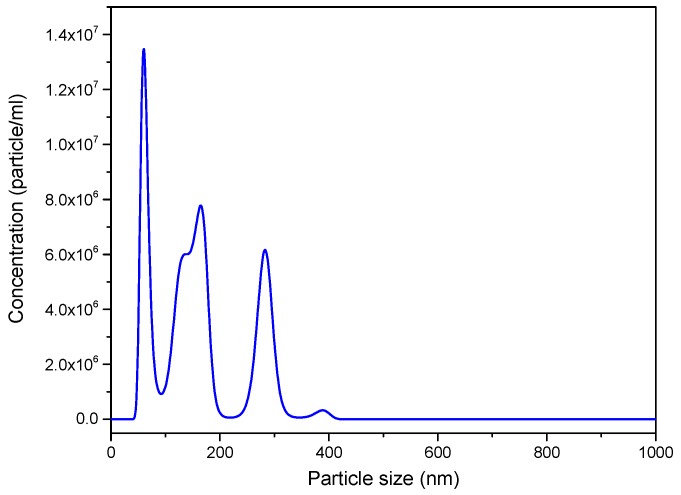
Nanoparticle tracking analysis (NTA) size distribution curve of GNS.

In order to further define the size of the graphene nanosheets, nanoparticle tracking analysis (NTA) was used (Figure 6). This technique provides individual particle sizing, intensity distribution and approximate concentrations (Figure S2). Different particle sizes ranging from 40 to 400 nm were observed. The average size of three measurements gave a value of 151.8 ± 12.7 nm and mean concentration of 1.02 × 10^9^ ± 6.48 × 10^7^ particles/g. However, most particles had sizes between ca. 30–200 nm, although the concentration of particles around 300 nm was also significant. The number of larger particles (>350 nm) was much lower. A comparison between DLS and NTA reveals that DLS could overestimate the particle sizes of the sample. Both techniques provide a hydrodynamic radius (R_h_) value but larger particles scatter stronger and are preferably detected by DLS than the smaller particles since the scattering intensity effect depends on size rather than on the number of particles with similar diameter. DLS is a fast, simple and non-destructive technique, but it presents this inherent feature when analysing samples that present some polydispersity [56]. In these cases, it shows distributions shifted to the largest particle sizes. NTA, on the other hand, distinguishes with a greater precision the distribution of sizes (10–2000 nm) because it is based on the tracking of individual particles (Figure S3) [27].

### 3.7. Particle Size Distribution by AF4

**Figure 7:**
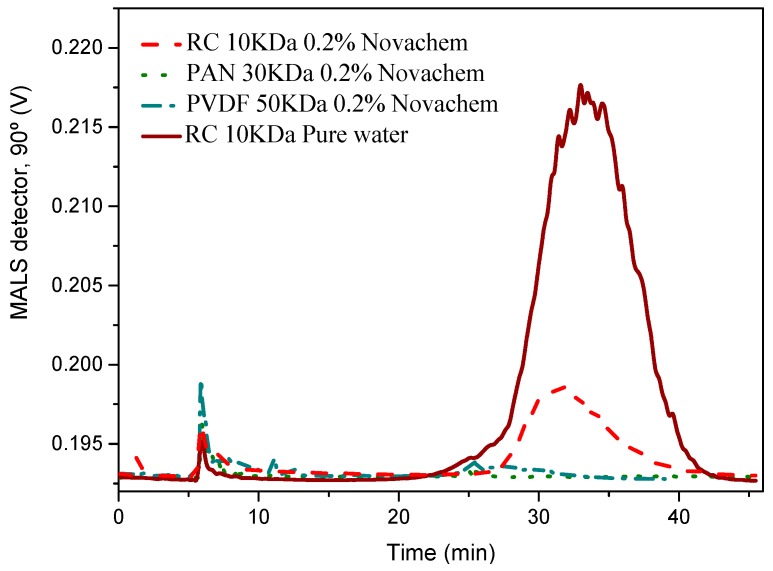
Asymmetric flow field flow fractionation (AF4) fractograms of GNS using regenerated cellulose (RC), polyacrylonitrile (PAN) and Polyvinylidene fluoride (PVDF) as membranes and Novachem and water as carriers.

Firstly, the influence of the membrane composition on retention properties and recovery from the AF4 channel was studied. Polyvinylidene fluoride (PVDF), polyacrylonitrile (PAN) and regenerated cellulose (RC) were used. Figure 7 shows the AF4 fractograms using different membranes and the frequently used 0.2% Novachem (Postnova Analytics) as carrier. Novachem is an aqueous surfactant mixture that stabilizes nanoparticles in solution.

The recovery from AF4 runs was determined as follows:

(5) $$R\left(\%\right)=\frac{S}{S_{0}}\cdot 100$$

where S and S_0_ are the peak areas without void peak of the detector signal (UV, λ = 254 nm/MALS 90°), obtained with and without cross-flow, respectively. The MALS detector allows the direct measurement of the radius of gyration in the 1–1000 nm particle size range [57].

The fractograms (90° light scattering detector) showed a low recovery using PAN and PVDF (less than 30%) due to the retention of graphene nanosheets in these membranes. It was easily visualized because they became black. This had to be due to the interaction of the graphene sheets with these polymeric membranes. However, the fractogram exhibited a broad peak centred at around 33 min using a RC with a recovery of 30%, thus suggesting a weaker interaction with this membrane. 

To increase the recovery, pure water was essayed as carrier. Figure 7 shows an AF4 fractogram of GNS using a RC membrane, which resulted in a recovery of 95%. These results indicated that there was no interaction between the membrane and the sample, and that water was the appropriate carrier to separate GNS by AF4.

**Figure 8:**
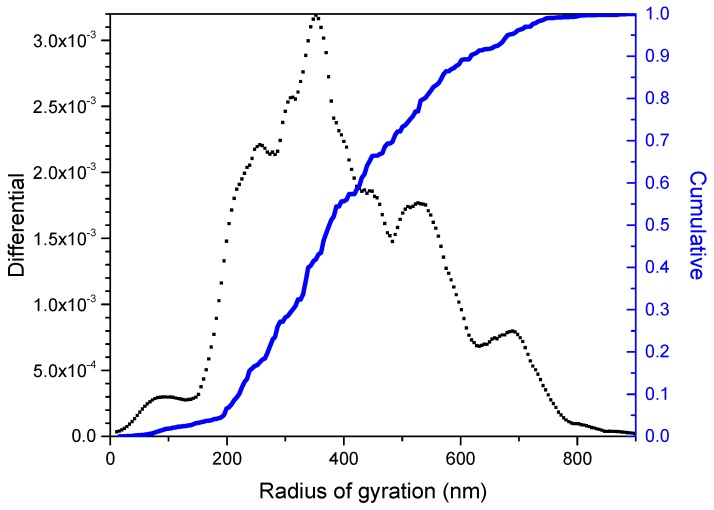
AF4 size distribution of GNS with a RC membrane using water as carrier.

The size distribution derived from the MALS detector showed the presence of different populations of particles (Figure 8). The most satisfactory fitting was achieved with the random coil model. This model is usually chosen for this particle range with all the different angles of the MALS detector providing robustness to the method (Figure S4). This detector provides the radius of gyration (R_g_), which is defined as the mass weighted average distance from the core of a particle to each mass element in the particle. R_g_ is more dependent on the structure of the particle than the value of R_h_. R_g_ increases with increasing the elution volume. Figure 8 shows the size distribution (radius of gyration in nm) as a function of the differential and cumulative distribution of the sample. The cumulative distribution indicates the fraction of a sample that has a mass in a certain range. Based on this, it is possible to determine GNS sample fractions simply by observing the height of the step. This parameter is provided by the differential distribution, which is calculated taking the differential of the cumulative distribution. In this way, it visually provides the weight fraction of the sample within a certain mass range, thus giving information on the resolution of the fractionation system and the analysis. The main peak of the fractogram was centred at around 360 ± 11 nm. As can be observed, this technique is able to discriminate populations of particles with similar sizes, thus allowing a better characterization of the graphene nanosheets. 

MALS detector coupled to AF4 can provide relevant information about the mechanism of aggregation and nanoparticle structure. This information is included in the fractal dimension structural parameter (D_f_). D_f_ gives a detailed information of the shape of the materials, thus allowing to understand the distribution of particles in aqueous media. A sphere has a D_f_ of 3, whereas D_f_ is 2 for a planar structure and 1 for a rigid rod [58,59]. In the case of GNS, the calculated value was approximately 2 (1.85), which corresponded to 2-D objects with plain surface (Figure S5), thus confirming the regular layer structure observed by TEM.

Also, additional information on the particle shape can be obtained by calculating the shape factor, p = R_g_/R_h_, where R_g_ is the average radius of gyration facilitated by AF4 (360 nm) and R_h_ is the Z-average hydrodynamic radius provided by DLS (450 nm). The theoretical value of p for a hard sphere is 0.778 and for a stiff rod of approximately 2 [60]. The shape factor increases as the particle deviates from an ideal homogeneous spherical shape to a prolate (p > 1) or oblate (0.775 < p < 1) ellipsoid [61,62]. The shape factor for GNS obtained by exfoliation of micrographite (MG) was 0.80, thus indicating that the GNS particles have an oblate ellipsoid shape. The ability of AF4 to separate and fractionate GNS particles can be used to minimize the polydispersity of graphene preparations and so to obtain more accurate data on size and shape of these materials in future investigations.

In summary, AF4 and NTA give much richer information on particle size than DLS when dealing with a polydisperse material as graphene nanosheets. AF4 can be considered a promising technique for the separation and characterization of a variety of graphene structures, including not only graphene nanosheets, as described in this work, but also graphene oxide, graphene quantum dots and other carbon nanostructures. Unlike DLS and NTA, AF4 is a separation technique and can be coupled to a great variety of detectors, thus gathering rich information from the sample. It separates particles in the range from 1 nm to 1–2 μm, although other field flow fractionation techniques could expand this range up to particle sizes higher than 100 μm. Interestingly, the existence of semipreparative channels would allow the separation and collection of milligram quantities of these graphene structures. In addition, NTA can provide the concentration of GNS nanoparticles. Nevertheless, stability concerns of aqueous dispersions of large graphene particles, a more limited range of particle size determination and the current limited availability in many laboratories are among the disadvantages of NTA and AF4 relative to DLS.

## 4. Conclusions

The ultrasound-assisted exfoliation of micrographite (MG) to graphene nanosheets (GNS) was confirmed by different techniques, such as XRD, TEM (sheets size between 200–500 nm) and Raman spectroscopy. The particle size distribution of GNS was firstly determined by DLS (ca. 450 nm), which provided the hydrodynamic radius and polydispersity of GNS. Subsequently, two different techniques, i.e., NTA and AF4, were applied here for the first time to graphene. NTA provided an average size of ca. 150 nm and higher resolution than DLS as well as approximate concentrations. In addition, it proved that DLS gave an average particle size whose intensity was biased towards the larger graphene particles. AF4 was able to separate various populations of GNS with different radius of gyration (centered at approx. 360 nm) and in addition it provided information concerning the structure and shape of the particles. Clearly, the application of NTA and AF4 besides DLS allows gathering much rich information on graphene particles. 

The above-mentioned advantages for NTA and AF4 reveal their usefulness for the determination of the actual polydispersity in graphene preparations, evaluation of their synthetic procedures and size determination and fractionation in aqueous media for studies related to biological applications, sensing and toxicity.

## Figures and Tables

**Table 1:** Analytical parameters and characteristics of asymmetric flow field flow fractionation (AF4).

AF4 Parameters	AF2000 System
Membranes	CR, PAN, PVDF
Channel geometry	Trapezoidal
Spacer thickness	350 µm
Focusing time	4 min
Elution time	40 min
Detector flow	0.5 mL/min
Injection flow	0.2 mL/min
Cross flow	1 mL/min
UV-Vis	254 nm

**Table 2:** Structural parameters of the studied materials derived from their XRD patterns.

Samples	Peak ° (2θ)	d_002_ (Å)	FWHM	Crystallite Size * (nm)
Micrographite	26.6	3.346	0.171	73.3
Graphene nanosheets	26.5	3.356	0.509	16.6

* Parameter calculated considering the instrumental broadening.

**Table 3:** Contribution of the six components used in fitting of the C 1s photoemission peak (in %).

Samples	C–C/C=C	C–O alkox	C–O epox	C=O	O–C=O	π→π*
(a) MG	65.7	16.9	6.4	4.3	3.9	2.8
(b) GNS	64.2	18.2	8.1	4.1	3.5	1.9

## Supplementary Materials

The following are available online at http://www.mdpi.com/2079-4991/9/2/152/s1, Figure S1: Calorimetric curve and linear fitting for the operational calibration of the ultrasonic equipment used for the GNS synthesis; Figure S2: NTA graph that represents GNS particle size and intensity; Figure S3: Nanoparticle tracking analysis images of GNS; Figure S4: Random Coil data fitting method; Figure S5: Analysis of the fractal factor dimension to identify the GNS particle shape where M is molecular weight and r is the radius of gyration.
