# Supplementary material for: A Comparative Study of Particle Size Distribution of Graphene Nanosheets Synthesized by an Ultrasound-Assisted Method
Source: Nanomaterials (Basel). 2019 Jan 26;9(2):152. doi: 10.3390/nano9020152 (PMC6409618; doi:10.3390/nano9020152)
Supplement: Supplementary file 1 [file nanomaterials-09-00152-s001.pdf]

Supplementary Information

A Comparative Study of Particle Size Distribution of Graphene Nanosheets Synthesized by An Ultrasound-Assisted Method

Juan Amaro-Gahete [1,†], Almudena Benítez [2,†], Rocío Otero [2], Dolores Esquivel [1], César Jiménez-Sanchidrián [1], Julián Morales [2], Álvaro Caballero [2,*] and Francisco J. Romero-Salguero [1,*]

[1] Departamento de Química Orgánica, Instituto Universitario de Investigación en Química Fina y Nanoquímica, Facultad de Ciencias, Universidad de Córdoba, 14071 Córdoba, Spain; q22amgaj@uco.es (J.A.-G.); q12esmem@uco.es (D.E.); qo1jisac@uco.es (C.J.-S.)

[2] Departamento de Química Inorgánica e Ingeniería Química, Instituto Universitario de Investigación en Química Fina y Nanoquímica, Facultad de Ciencias, Universidad de Córdoba, 14071 Córdoba, Spain; q62betoa@uco.es (A.B.); b42otizr@uco.es (R.O.); iq1mopaj@uco.es (J.M.)

* Correspondence: alvaro.caballero@uco.es (A.C.); qo2rosaf@uco.es (F.J.R-S.); Tel.: +34957218620 (A.C.)

† These authors contributed equally to this work.

A calorimetric method was applied for the calibration of the Ultrasonic Homogenizer 4710 Series from the Cole Parmer Instrument Co., following the method described by Taurozzi et al. [1]. The experimental procedure to carry out the calibration was:

- Fill a 100 mL beaker with 50 mL of o-dichlorobenzene (ODCB), determining the mass of this solvent (65.11 g).
- The ultrasound probe (Ultrasonic Homogenizer 4710 Series from Cole Parmer Instrument Co.) is introduced under the surface of the solvent approximately 2.5 cm. Then, a thermometer is introduced approximately 1 cm below the tip of the ultrasonic probe.
- The conditions of the ultrasound equipment are selected by setting 40% amplitude, 60% duty cycle and pulsed mode of operation.
- Temperature and time data are recorded in order to carry out a linear fitting by least-squares regression.

Following the next equation: $P = \frac{dT}{dt} M C_p$

$C_{p,\,ODCB} = 170.68\ J \cdot mol^{-1} \cdot K^{-1} = 1.219\ J \cdot g^{-1} \cdot K^{-1}$; $dT/dt = 0.12545\ K/s$

The resulting power value was P = 9.96 W.

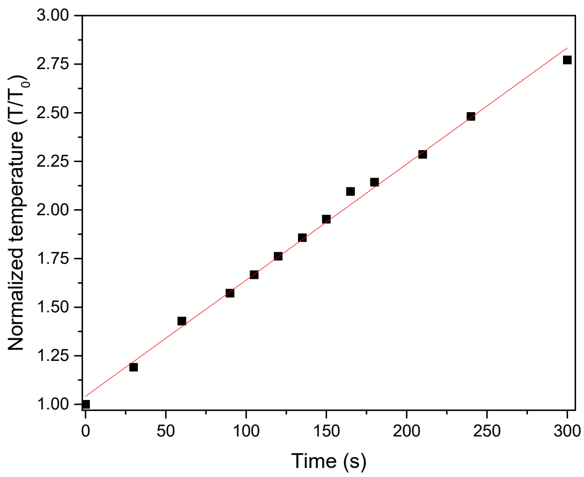

Figure S1. Calorimetric curve and linear fitting for the operational calibration of the Ultrasonic equipment used for the GNS synthesis.

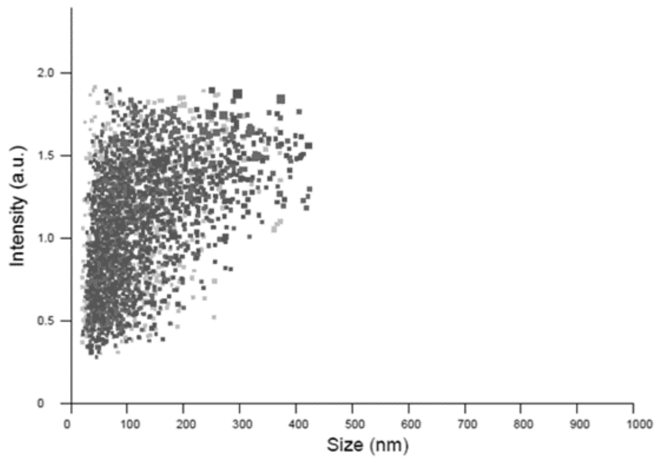

Figure S2. NTA graph that represents GNS particle size and intensity.

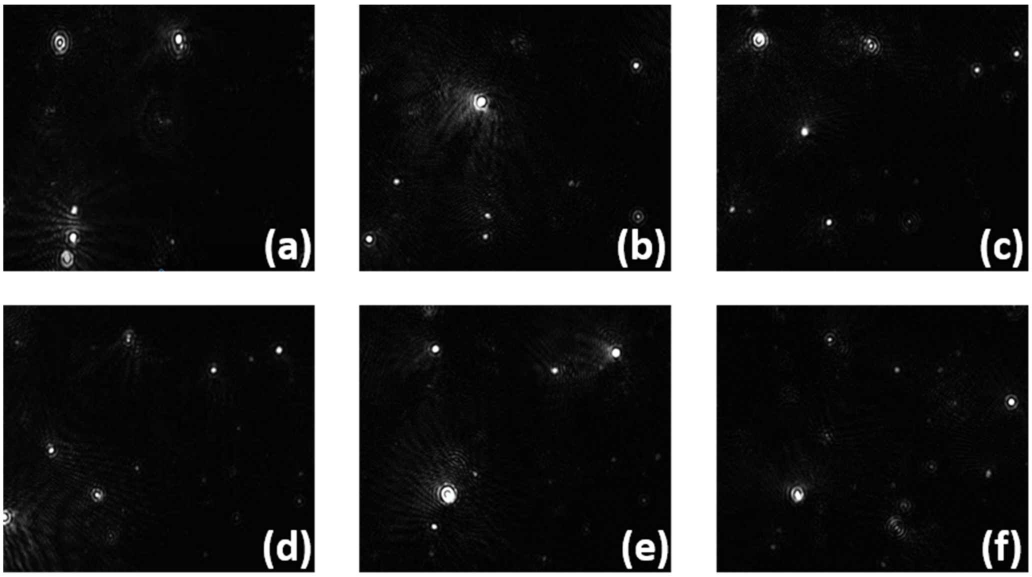

Figure S3. Nanoparticle tracking analysis images of GNS (a–f).

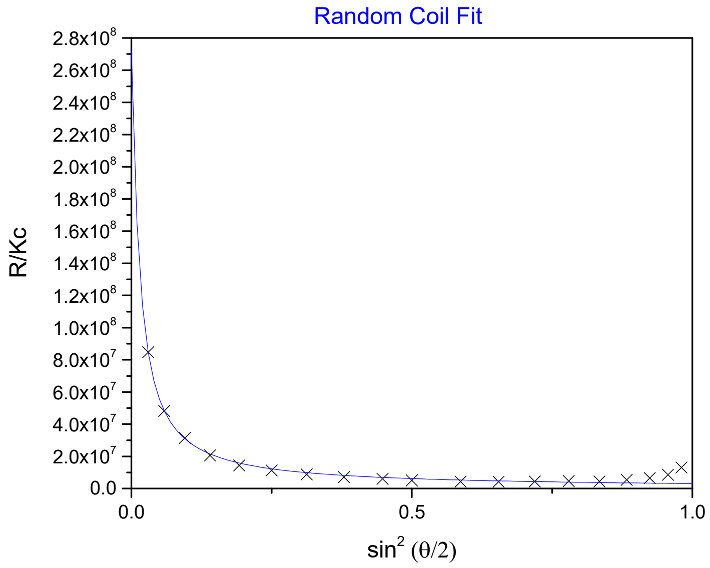

Figure S4. Random Coil data fitting method.

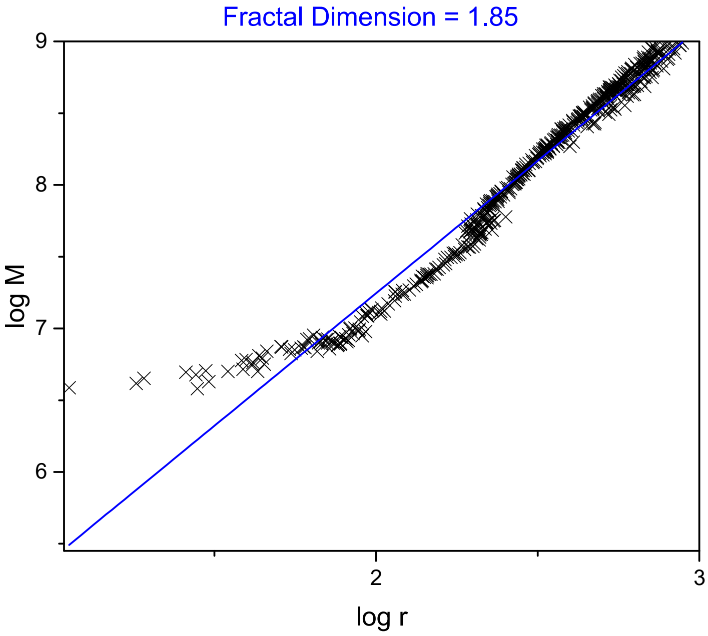

Figure S5. Analysis of the fractal factor dimension to identify the GNS particle shape where M is molecular weight and r is the radius of gyration.

References

1. Taurozzi, J.S.; Hackley, V.A.; Wiesner, M.R. Ultrasonic dispersion of nanoparticles for environmental, health and safety assessment—issues and recommendations. *Nanotoxicology* **2011**, *5*, 711–729, doi:10.3109/17435390.2010.528846.
